# Epigenetic Regulators of DNA Cytosine Modification: Promising Targets for Cancer Therapy

**DOI:** 10.3390/biomedicines11030654

**Published:** 2023-02-21

**Authors:** Inkyung Jung, Jungeun An, Myunggon Ko

**Affiliations:** 1 Department of Biological Sciences, Ulsan National Institute of Science and Technology, Ulsan 44919, Republic of Korea; 2 Department of Life Sciences, Jeonbuk National University, Jeonju 54896, Republic of Korea; 3 Center for Genomic Integrity, Institute for Basic Science, Ulsan 44919, Republic of Korea

**Keywords:** epigenetics, DNMT, TET dioxygenases, DNA methylation and demethylation, small molecules, cancer therapy

## Abstract

Epigenetic modifications are crucial regulators of gene expression that critically impact cell lineage differentiation, survival, and proliferation, and dysregulations are commonly observed in various cancers. The aberrantly modified epigenome confers unique features on tumor cells, including sustained proliferative potential, resistance to growth-suppressive or cell death signals, augmented replicative immortality, invasion, and metastasis. As a result, epigenetic abnormalities exhibit significant impacts on all stages of oncogenesis from its onset to progression to metastasis. Among various epigenetic mechanisms in mammals, DNA cytosine methylation–demethylation is recurrently disrupted in cancers. Due to its inherent reversibility, targeting DNA methylation dynamics has gained tremendous attention as a promising therapeutic option that can ameliorate the effects of cancer-specific epigenetic abnormalities by restoring normal conditions. Various small molecules targeting DNA (de)methylation regulators have been developed as potential cancer therapeutics, some of which are approved for usage in clinics. Clinical trials of many other molecules are underway for both hematological malignancies and solid tumors. In this review, we discuss the DNA methylation/demethylation pathway as a promising target for therapeutic intervention in cancer and highlight the development of various epigenetic drugs targeting DNA-modifying enzymes such as DNA methyltransferases (DNMTs) and ten-eleven translocation (TET) enzymes.

## 1. Introduction

In eukaryotes, genomic DNA is condensed into chromatin by wrapping around histone octamers in the nucleus. Euchromatin has an open structure and encompasses the majority of transcriptionally active genes. Conversely, a highly condensed chromatin structure termed heterochromatin usually contains silent genes and is important for guarding genomic integrity by protecting the genome from transposon activation and exerting key regulatory functions. Epigenetics is defined by a heritable genomic mechanism that reversibly influences gene expression without changes in DNA sequences [1,2]. Chromatin structure and accessibility to transcription factors are dynamically regulated by epigenetic mechanisms such as histone modification, DNA methylation, and nucleosome mobilization.

The two best-studied epigenetic modifications are DNA and histone modifications [1,2], which are catalyzed by “writers”, including DNA methyltransferases (DNMTs), histone methyltransferases (HMTs), histone acetyltransferases (HATs), and E3 ubiquitin ligases. DNMTs catalyze the transfer of a methyl group to cytosine residue in DNA, while HMTs induce post-translational modification by adding a methyl group to lysine residues on histones. Moreover, HATs transfer acetyl groups from acetyl-CoA donors to the amino group of lysine residues on nucleosomal histones or newly translated non-nucleosomal histones. “Readers” recognize and interpret specific epigenetic marks on DNA and histones. Methyl CpG binding proteins (MBPs), including methyl-CpG-binding domain (MBD)-containing proteins, the Set- and RING-associated (SRA) domain-containing proteins, and methyl-CpG-binding zinc finger proteins, can recognize DNA methylation [3]. Histone methylation readers specifically recognizing methylated lysine residues on histones include various proteins such as PHD finger protein 1 (PHF1), retinoblastoma protein-binding protein, and Bromodomain PHD finger transcription factor (BPTF) [4,5,6]. Lastly, “erasers” remove epigenetic modifications, resulting in a change in gene expression patterns [7]. DNA methylation and demethylation are catalyzed by the two enzymes, DNMT and TET proteins. Once DNMTs generate 5mC marks on DNA, TET proteins remove the methyl group from 5-methylcytosine [8]. Moreover, the demethylation of lysine residues on histone proteins is catalyzed by various histone lysine demethylases (HDMs) such as lysine demethylase 1A (LSD1/KMD1A), 5B (KDM5B), and JmjC domain-containing proteins [9,10]. On the other hand, histone deacetylases (HDACs) remove acetyl groups from histones, resulting in a compact transcriptionally repressive chromatin organization. HDACs are classified as class I, II, III, or IV; classes I, II, and IV are Zn^2+^-dependent, while class III is nicotinamide adenine dinucleotide (NAD)-dependent [2].

Above all, DNA methylation/demethylation is one of the most thoroughly studied epigenetic mechanisms. In mammals, the DNA methylation/demethylation balance directly regulates essential biological processes such as gene expression, chromatin organization, DNA imprinting, and X-chromosome inactivation; thus, it coordinates critical events including embryonic development and cellular differentiation and function [1]. Importantly, the maintenance of DNA methylation/demethylation balance is also critical for maintaining genomic stability. The disrupted DNA methylation status is associated with increased genomic instability that can lead to malignant transformations [11]. Global DNA hypomethylation and locus-specific focal hypermethylation are epigenetic hallmarks of cancer. DNA hypomethylation at repetitive regions across the genome disrupts the structure and normal physiological function of heterochromatin and derepresses transposable elements, facilitating genome instability that can directly contribute to tumorigenesis [12]. Furthermore, localized hypermethylation at the promoter region of tumor suppressor or repair genes can induce heritable transcriptional silencing [13]. For instance, hypermethylation at the promoter region of the *p16* tumor suppressor gene frequently occurred in lung cancers [14], and a DNA repair enzyme, *MGMT*, was also frequently inactivated by promoter hypermethylation in brain tumors [15].

In this review, we introduce the mechanisms through which DNA methylation/demethylation influences cancer development, highlighting the development of various DNMT-targeting anti-cancer therapeutics over the last decades and their effectiveness in clinical treatment. Furthermore, we also discuss the key findings supporting the potential of various small molecules targeting TET-family dioxygenases in cancer therapy, including both the agonists and antagonists of TET enzymes.

## 2. DNA Methyltransferases and Their Inhibitors

### 2.1. The Role of DNA Methyltransferases

**Figure 1:**
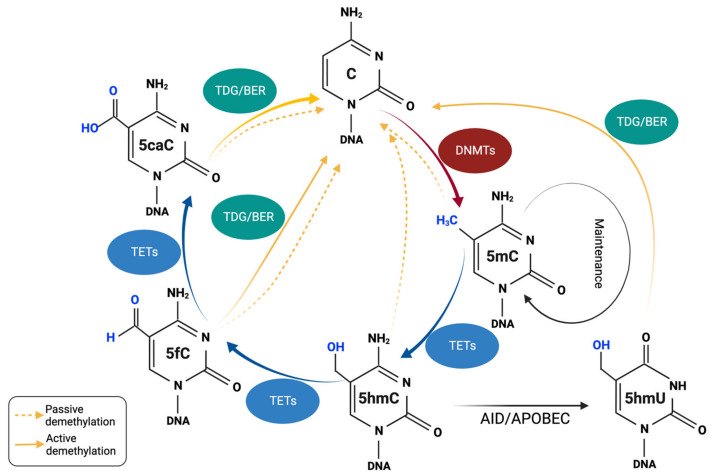
DNA methylation and demethylation mechanisms. DNA methyltransferases (DNMTs) add a methyl group to the C5 position of cytosines (C) to form 5-methylcytosines (5mC). DNMT3A and DNMT3B are responsible for de novo methylation during development, while DNMT1 is involved in maintaining DNA methylation in the newly replicated DNA strand. DNA methylation can be reversed passively via replication-dependent dilution. Active DNA demethylation processes can also occur by the action of ten-eleven translocation (TET) proteins that successively oxidize 5mC to oxidized methylcytosines (oxi-mCs), including 5-hydroxymethylcytosine (5hmC), 5-formylcytosine (5fC), and 5-carboxylcytosine (5caC). Of these, 5hmC can be deaminated to 5-hydroxymethyluracil (5hmU); 5fC, 5caC, and 5hmU are replaced by an unmodified cytosine via thymine DNA glycosylase (TDG)-mediated base excision repair (BER). Created with BioRender.com (accessed on 20 January 2023).

DNA methylation occurs at the 5-carbon (C5) position of cytosine, yielding 5-methylcytosine (5mC), and occurs predominantly at cytosine in the context of the cytosine–guanine (CpG) dinucleotide [16]. The majority of CpG dinucleotides are concentrated within CpG-rich DNA regions referred to as CpG islands that are mostly located near transcription start sites. The hypermethylation of CpG islands often leads to the silencing of the genes by prohibiting the binding of some transcription factors or recruiting methyl-CpG-binding proteins that interact with repressive histone-modifying enzymes [17]. DNA methyltransferases (DNMTs), including DNMT1, DNMT3A, and DNMT3B, are responsible for the transfer of a methyl group from the methyl donor S-adenosyl-L-methionine (SAM) to cytosine (Figure 1). The most abundant DNMT, DNMT1, preferentially maintains parental methylation patterns during replication and cell division [18,19] while DNMT3A and DNMT3B are responsible for de novo methylation that target unmethylated CpG to establish new DNA methylation patterns [20].

### 2.2. Impact of DNMT Aberrations on Cancers

Both genetic or epigenetic alterations caused by intrinsic or extrinsic factors can alter the expression or function of DNMTs, which results in genome-wide methylation changes and aberrant gene expression [21]. In murine embryonic stem cells (mESCs), a deficiency of DNMTs resulted in a global change in DNA methylation. DNA methylation is essential for mice survival, as shown by increased embryonic lethality in DNMT1 or DNMT3B single knockout mice, and early postnatal lethality in DNMT3A single knockout mice [20,22,23,24]. In addition, in DNMT triple-knockout mESC, a reduction in the methylation and the preservation of stem cell characteristics and self-renewal were observed [25]. Notably, the dysregulation of DNMT, as a consequence of gene mutations or altered expression, is closely associated with the onset and/or progression of various types of tumors in the lung, breast, stomach, and colon, as well as hematologic cancers such as leukemia [26,27,28]. For instance, *DNMT1* mutations have been described in colorectal cancer [29], and the disrupted activity of DNMT1 in mice was reported to induce repetitive insertions of a transposable element within the *Notch* gene leading to its oncogenic activation in thymic lymphomas [26]. In addition, loss-of-function mutations in the *DNMT3A* gene are one of the most frequent defects identified in acute myeloid leukemia (AML; ~30%) or myelodysplastic syndrome (MDS; 2–10%) patients [30]. Intriguingly, over half of *DNMT3A* mutations occur at the R882 amino acid residue in AML patients. Germline mutations in *DNMT3B* cause immunodeficiency, centromeric instability and facial anomalies (ICF) syndrome and chromosome instability, and single nucleotide polymorphisms in *DNMT3B* are associated with an increased risk of several cancers including breast and lung adenocarcinoma [31,32]. In contrast, the overexpression of DNMTs was also observed across various types of cancers. DNMT1 overexpression was found in pituitary adenoma, pancreatic cancers, gastric cancers, lung cancers, and thyroid cancers [21]. In particular, in gastric cancers, the overexpression of DNMT1 correlated with poor tumor differentiation and caused the hypermethylation of multiple CpG islands, including those of *p16*, *hMLH1*, and *E-cadherin* [33]. In lung cancers, DNMT1 overexpression led to the hypermethylation and dysregulation of multiple tumor suppressor genes, including *p16*, *RARβ*, *FHIT*, and *RASSF1A* [34]. DNMT3A was also overexpressed in many human cancers such as pituitary adenoma, acute myeloid leukemia, and vulvar squamous cell carcinoma and led to the silencing of tumor suppressors such as p16 and RASSF1A [21]. Likewise, DNMT3B overexpression is frequent in lung cancer, hepatocellular carcinoma, ovarian cancer, and breast cancer, resulting in the hypermethylation of multiple genes [21]. As described earlier, the global hypomethylation commonly observed in cancers can contribute to oncogenesis. More importantly, the hypermethylation of CpG islands in gene promoters leads to the silencing of various tumor suppressor and repair genes and has profound effects on key cancer-associated cellular processes, including cell cycle progression, apoptosis, DNA repair, and angiogenesis. Thus, the ability of hypomethylating agents to reactivate silenced genes via the reversal of DNA hypermethylation primarily underlies DNMT-targeting cancer therapies. In Section 2.3, we will explore the current status of development, clinical utility, and challenges with respect to various therapeutically applicable DNMT inhibitors.

### 2.3. DNMT Inhibitors

Over the past decades, several DNMT inhibitors (DNMTis) have been proposed to treat cancers (Table 1) [35,36]. The first two compounds identified as DNMTis were the cytosine analogs 5-azacitidine (vidaza, AZA) and 5-aza-2′-deoxycytidine (decitabine, DEC). These azanucleosides substitute nitrogen for the carbon at the C5 position of the pyrimidine ring. Upon incorporation into the DNA double helix, they bind to DNMT irreversibly, presumably resulting in DNMT degradation and DNA demethylation [37]. Indeed, decitabine was shown to decrease both DNMT1 and DNMT3A expression, while azacitidine only targets DNMT1. Without DNMTs, CpG sites lose their methylation after cell replication, and the transcription of the genes that were previously silenced by promoter methylation may be derepressed.

DNMTis can synergistically activate tumor suppressor genes when treated in combination with HDAC inhibitors [58]. Azacitidine and decitabine were approved by the US Food and Drug Administration (FDA) for the treatment of MDS in 2004 and 2006, respectively, constituting the core therapy for the disease so far, and clinical trials are ongoing for other hematologic malignancies and solid tumors with single agents or in combination therapies [38,42]. Although DNMTis are widely used in the treatment of AML and MDS, they still have limitations. First, a high recurrence rate was observed in clinical trials in MDS patients after treatment with DNMTi. Next, a tolerance issue has been raised as drug sensitivity decreases remarkably in the case of continuous DNMTi treatments due to resistance to drugs [59,60]. Furthermore, cytidine deaminase expressed in multiple organs in the body seemed to shorten the plasma half-lives of the first-generation DNMTi.

To overcome these limitations, recent studies and clinical trials have addressed the possibility of developing more stable and effective DNMTi, modifying treatment regimens, and combining them with other drugs. Researchers have developed guadecitabine (SGI-110) as a second-generation hypomethylating agent; its active metabolite is decitabine, which displays improved stability, enhanced DNA incorporation in dividing cells, and more importantly augmented resistance to degradation by cytidine deaminase [61], leading to prolonged half-life and enhanced efficacy in MDS and AML, compared with intravenous decitabine. Additional drugs including 4′-thio-2′-deoxycytidine (TdCyd) and 5-fluoro-2′-deoxycytidine (FdCyd) have been assessed under clinical trials for the treatment of solid tumors and some hematologic neoplasms [46]. TdCyd is thought to display its effects by being incorporated into DNA where it engages the active site of DNMT1, offering an improvement over current DNMTi mainly due to its higher incorporation rate into DNA at lower levels of cytotoxicity. FdCyd normally has a very short half-life (~10 min) due to its susceptibility to cytidine deaminase. Thus, clinical trials of the combined treatment of this drug with tetrahydrouridine (THU), an inhibitor of cytidine/deoxycytidine deaminase, are underway to assess its anti-cancer potency.

Furthermore, as another route to improve the functionality of drugs, including bioavailability, chemical stability under physiological conditions, and higher selectivity, non-nucleoside analogs have also been developed, which include DNA binders, oligonucleotides, SAM antagonists, natural compounds, and repurposed drugs [62,63,64]. These molecules mainly act as DNMT inhibitors by binding to the catalytic site of DNMT and interfering with the interaction with DNA. Among the non-nucleoside DNMTi, procainamide and its analog procaine have been approved by the FDA and are used as anti-arrhythmic drugs and local anesthetics, respectively. They were shown to bind to DNA in cancer cells, reducing methylation levels [65]. Nanomycin A is also known as a DNMT3B-specific epigenetic inhibitor, and the arterial vasodilator hydrazine has also been found to inhibit DNA methylation, inducing genomic demethylation [50]. Nevertheless, since these compounds generally have the disadvantage of low specificity, the development of compounds that specifically target DNA methylation machinery is necessary. As an example, MG98 was an anti-sense oligonucleotide targeting *DNMT1* mRNA, effectively reducing DNMT1, but its clinical effectiveness is controversial [54,66]. Most recently, a reversible DNMT1-specific inhibitor (known as GSK3685032) was shown to compete with the active-site loop of DNMT1 for penetrating hemimethylated DNA. As a result, it induced profound DNA hypomethylation, transcriptional activation, and the inhibition of cancer cell growth. Importantly, GSK3685032 displayed improved in vivo tolerability relative to decitabine, thus providing greater tumor regression and better survival in the murine models of AML [57]. Taken together, a wide range of DNA hypomethylating agents, including cytidine analogs and non-cytidine analogs, has been developed, and they exhibit clinical benefits in hematologic and solid cancers. However, many of them induce substantial cytotoxicity to normal cells, limiting their clinical utility. Thus, a deeper understanding of the molecular basis of various DNMTi actions and the mechanisms underlying drug resistance will facilitate the development of more effective DNMT-targeted epigenetic therapies alone or in combination with conventional therapies, in addition to patient-specific customized therapy by choosing the DNMTis that work best for each patient.

## 3. DNA Demethylation and TET Proteins

### 3.1. TETs and the Mechanisms of Passive/Active DNA Demethylation

DNA methylation is a stable and highly conserved epigenetic mark in many organisms and significantly impacts different biological processes, including genomic imprinting, X-chromosome inactivation, and the suppression of transposons [67,68]. The mechanism of the replication-dependent dilution of 5mC in cells with defective DNMT1 has been well defined. However, how methyl groups are lost independently of DNA replication has remained a mystery until the discovery of the function of TET-family 5mC oxidases, which consist of three members: TET1, TET2, and TET3 [69]. *TET1* was the first member identified as a fusion partner of the mixed lineage leukemia (MLL) gene in patients bearing a ten-eleven chromosomal translocation t(10;11)(q22;q23) [8]. Based on the sequence homology, two additional *TET* genes, *TET2* and *TET3* were identified in the human genome. A series of experiments has shown that TET1 oxidizes 5mC to 5-hydroxymethylcytosine (5hmC) [69] and subsequent research demonstrated that TET2 and TET3 can also catalyze 5mC oxidation. All three TETs are capable of further oxidizing 5hmC to 5-formylcytosine (5fC) and 5-carboxylcytosine (5caC) (Figure 1) [70]. The TETs’ enzymatic activity critically relies on iron (Fe^2+^) and α-ketoglutarate (α-KG, also known as 2-oxoglutarate) [71,72]. α-KG is an intermediate metabolite produced in the tricarboxylic acid (TCA) cycle: Isocitrate dehydrogenases (IDHs: IDH1 and IDH2) catalyze the oxidative decarboxylation of isocitrate to produce α-KG. Congruent with this, withdrawing Fe^2+^ and α-KG significantly impaired TET enzyme functions. Moreover, mutations at the Fe^2+^- or α-KG-binding sites in the active site of TET dioxygenases almost completely abolished the 5hmC signal in the cultured cells [73].

The modification of 5hmC is stable, but its levels vary depending on tissue types, corresponding to 5–10% of the total 5mCs in ESCs and 40% in Purkinje cells in the brain [69,74,75]. Replication-dependent “passive” DNA demethylation can occur when the parental strand harbors 5hmC during DNA replication cycles due to the low affinity of DNMT1 for the hemi-5hmC site compared to the hemi-5mC site (Figure 1) [76,77,78]. Therefore, multiple rounds of DNA replication can lead to the progressive dilution of cytosine methylation, thus called passive demethylation. In addition, 5fC and 5caC can be excised by the DNA repair enzyme thymine DNA glycosylase (TDG), and they can be replaced by an unmodified cytosine via base excision repair (BER), which is termed “active” demethylation (Figure 1) [13,79]. It has been shown that 5hmC is deaminated to 5-hydroxymethyluracil (5hmU), followed by conversion to cytosines by virtue of the TDG/BER pathway. It was also shown that TDG targets 5fC:G and 5caC:G with higher affinity than thymidine or 5hmC:G mismatches. The loss of TDG in ESCs has shown that TDG deficiency increased 5fC and 5caC levels up to 10-fold [80]. Moreover, other components of the DNA damage and BER pathway, including p53, PARP, GADD45, and NEIL1/2, have been targeted 5fC or 5caC and prevent DNA hypermethylation [81,82,83]. TET proteins were proposed to oxidize thymine to 5hmU, causing mismatched 5hmU:A and resulting in the indirect removal of 5mC from the genome by a subsequent long patch BER or non-standard mismatch repair pathway [84,85]. Taken together, these results indicate that the balance between DNA methylation and demethylation in mammals is dynamically modulated by the combined actions of DNMTs and TET enzymes.

### 3.2. LOF of TET and Hematopoietic Cancer

Compared with normal cells, a wide range of cancer cells display significantly low or undetectable 5hmC levels; thus, the loss of 5hmC is considered a novel epigenetic hallmark of human cancer. Previous studies have shown that the loss of TET function is commonly observed across multiple solid tumors and blood cell malignancies, which can be induced by various mechanisms, including genetic mutations in the coding region of *TET* genes or other ways without involving coding region mutations [86,87,88]. Initially, the possible involvement of TET dysregulation in tumorigenesis was suggested by identifying TET1 as a fusion partner of MLL in patients with AML, which was also noticed in T cell lymphoma and B cell acute lymphoblastic leukemia [89,90]. TET1 enhanced global 5hmC levels in MLL-rearranged ALL. It was a downstream target of MLL fusion proteins and regulates the expression of critical cancer-associated genes such as *HOXA9, MEIS1*, and *PBX3* [91,92]. Since then, it has been reported that *LOF* mutations in *TET2* genes are prevalent in hematopoietic malignancies, although mutations in *TET1* and *TET3* are rare [88,93,94,95]. *TET2* LOF mutations were found in ~20% of MDS patients, ~20% of myeloproliferative neoplasms (MPN), ~20% of AML, and ~45% of chronic myelomonocytic leukemia (CMML). It was also reported that myeloid malignancies harboring *TET2* mutations exhibit substantially reduced levels of 5hmC compared with wild-type samples [75,96]. Consistent with these observations, various murine models deficient for TET proteins in the hematopoietic system developed various types of hematologic malignancies, including AML, MPN, MDS, and lymphomas (extensively reviewed recently in [97]). Interestingly, *TET2 LOF* mutations were mutually exclusive with *IDH1/IDH2* gain-of-function mutation, which is also known to induce DNA hypermethylation and inhibit differentiation in hematopoietic cells [96]. There are mutational hotspots for *IDH* genes: Most *IDH1* mutations occur in the R132 amino acid residue, and *IDH2* mutations are frequent in the R140 and R172 residues [98]. Somatic *IDH* mutations are neomorphic mutations that convert α-KG to oncometabolite (*R*)-2-hydroxyglutarate (2HG), which suppressed the conversion of 5mC to 5hmC via impeding α-KG association with TET enzymes [99,100,101]. Mutations in IDH1 and IDH2 are also common in gliomas, AML, chondrosarcomas, and cholangiocarcinoma. In addition to mutations in TETs and IDHs, a decreased expression of TETs and IDHs was also involved in the loss of 5hmC in cancer [102]. In chronic lymphocytic leukemia, the reduced expression of TET1 and TET3 was observed with the low expression of the IDH1 in leukemic B cells compared with normal healthy B cells, which occurred due to the decrease in α-KG.

### 3.3. TET Deficiency and Solid Cancers

It has also been reported that TET proteins also play important roles in solid cancers, generally acting as tumor suppressors. Reduced levels of TETs have been associated with decreased 5hmC levels in human prostate, lung, breast, liver, and pancreatic cancerous tissue compared with non-cancerous tissue [87]. In early-stage breast cancer patients, low levels of *TET1* mRNA were associated with poor survival rates, and in breast cancer cell line and mouse xenograft models, TET1 was reported to regulate tumor growth and metastasis by demethylating *HOXA7* and *HOXA9* promoter regions. The anti-tumorigenic role of TET1 has also been reported in colorectal cancer. TET1 and 5hmC were downregulated in primary colon cancer compared with wild-type surrounding tissues and inhibited tumor growth by derepressing inhibitors of the WNT pathway [103]. A low level of TET1 expression was also associated with poor survival in pancreatic cancer patients. In pancreatic cancer cells, TET1 inhibited tumor growth by suppressing the WNT/β-catenin pathway by elevating the expression of SFRP2, an upstream inhibitor of the WNT/β-catenin pathway, via enhanced 5mC oxidation at its promoter [104]. In addition, TET2 reduction and 5hmC levels were associated with high tumor grade, pathologic stage, metastasis, and vascular thrombosis in 130 ovarian carcinoma patients [105]. TET3 was also shown to suppress TGF-β1-induced epithelial–mesenchymal transition (EMT) by demethylating the *miR-30d* precursor gene in ovarian cancer cells [106]. Moreover, in melanoma cells, by sensing the TGF-β signal, DNMT3A was recruited to *TET2* and *TET3* promoters to silence their expression and thereby promoted an EMT-like process [107]. As mentioned earlier, TET enzymes are key modulators of genes involved in various biological pathways. In particular, the silencing of TET proteins induces abnormal methylation of tumor suppressor genes, resulting in tumor formation, cell proliferation, migration, and invasion. Therefore, restoring TET and 5hmC levels may be a promising therapeutic strategy for the treatment of a broad range of cancers.

## 4. TET Modulators and Cancer Therapy: Role of Vitamin C

### 4.1. Biological Functions of Vitamin C

**Figure 2:**
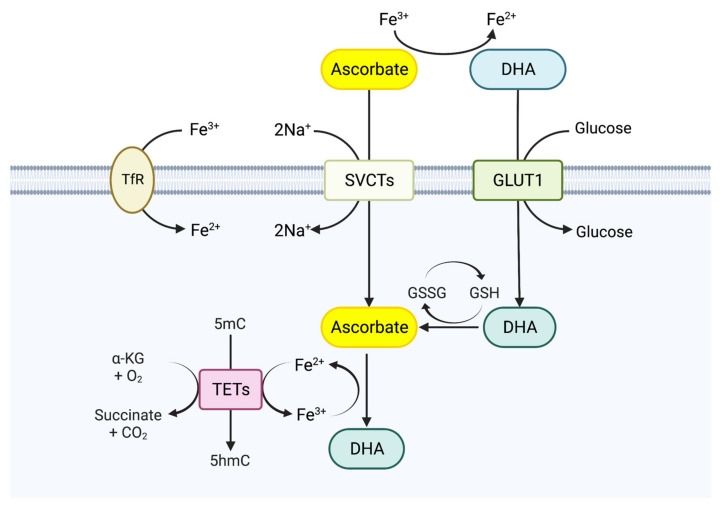
Vitamin C uptake and its function as a cofactor for TET proteins. The reduced form of vitamin C, ascorbate, is transported into the cells via sodium-dependent vitamin C transporters (SVCTs; SVCT1/SVCT2), whereas its oxidized form, dehydroascorbic acid (DHA), is transported into the cells via glucose transporters such as glucose transporter 1 (GLUT1). Upon entry into cells, DHA is reduced back to ascorbate. Vitamin C promotes the reduction of Fe^3+^ to Fe^2+^, thereby functioning as a cofactor for the TET enzyme that promotes the TET-mediated demethylation pathway. GSH, glutathione; GSSG, glutathione disulfide; TfR, transferrin receptor. Created with BioRender.com (accessed on 20 January 2023).

Vitamin C is present in plasma mainly in its reduced form (ascorbate, ASC) at a concentration ranging from 5 to 10 μM. Ascorbate is transported into the cytosol mainly through two sodium-dependent vitamin C transporters (SVCTs; SVCT1 and SVCT2), which are also known as members of the solute carrier gene family 23 (SLC23). Vitamin C can also be actively transported in its oxidized form (dehydroascorbate, DHA) through glucose transporters (GLUTs; GLUT1, GLUT3, and GLUT4) (Figure 2) [108,109]. Inside cells, glutathione (GSH) rapidly reduces DHA back to ASC. At lower levels, vitamin C exhibits its effect as an antioxidant to reduce harmful reactive oxygen species at low concentrations. However, at high plasma concentrations, it can function as a pro-oxidant [110,111]. Vitamin C enhances the activity of diverse Fe (II)- and α-KG-dependent dioxygenases, including TET proteins, histone lysine demethylases (KDMs), and hypoxia-inducible factor (HIF) prolyl hydroxylases (Figure 2) [111]. During somatic cell reprogramming, vitamin C was shown to enhance the removal of histone modifications by KDM5 and KDM2 [112]. Moreover, in osteosarcoma (U2OS) cells, vitamin C increased the ability of KDM5B/C to demethylate H3K4me3, and KDM5B has been shown to increase DNA double-strand break repair by recruiting factors Ku70 and BRCA1 [112,113]. In addition, treatment with vitamin C could promote the degradation of HIF-1α by stimulating HIF prolyl hydroxylases, and growth-inhibitory effect of vitamin C in leukemia cell cultures correlated with the downregulation of HIF-1α expression [114,115]. The stabilization of HIF was also related to resistance to DNA damage. Hypoxic mouse stromal cells were more resistant to radiation than normal-state stromal cells, and lung cancer cells with increased miRNA expression patterns by HIF1 stabilization exhibited a phenotype similar to a hypoxic state and increased radioresistance [116,117]. Lastly, vitamin C treatment has been shown to reduce cancer cell proliferation in the mutant IDH1-related AML model, in which demethylation occurred in the loci of myeloid differentiating factors, indicating that vitamin C treatment can induce epigenetic reprogramming and offset the effect of 2HG in the TET demethylase activation pathway [118].

### 4.2. TET Enzymes and Vitamin C

**Figure 3:**
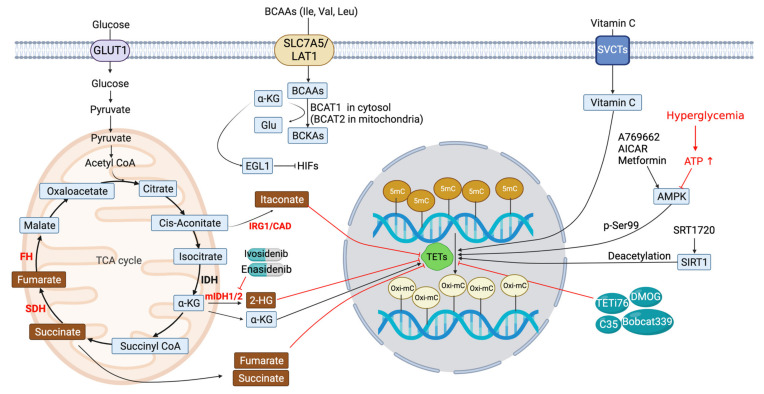
Mechanism of action of the known TET activators and inhibitors. TETs and 5hmC have a crucial role in tumorigenesis and metastasis. Targeting TET protein function presents promising and specific strategies for anti-cancer therapy. TETs have multifaceted roles in different cancers, mainly exerting their effects as tumor suppressors. The impact of both TET agonists (e.g., mutant IDH inhibitors, vitamin C, AMPK agonist, and SIRT1 agonist) and antagonists (2HG, succinate, fumarate, itaconate, and newly identified small molecules) of TET proteins on cancer treatment are actively studied in vitro and in vivo. IRG1, immune response gene 1; CAD, cis-aconitic acid decarboxylase; IDH, isocitrate dehydrogenase; SDH, succinate dehydrogenase; FH: fumarate hydratase; LAT1, L-type amino acid transporter; BCAAs, branched-chain amino acids; BCKA, branched-chain α-keto acids; SVCT, sodium-dependent vitamin C transporter; AMPK, AMP-activated kinase; SIRT1, Sirtuin 1. Created with BioRender.com (accessed on 20 January 2023).

Many studies have shown that vitamin C augments 5mC oxidation and DNA demethylation by stimulating TET enzymes via the enhanced reduction of Fe^3+^ to Fe^2+^ (Figure 3). Studies using ESCs and fibroblasts confirmed that vitamin C improves the quality of cultured cells and promotes induced pluripotent stem cell (iPSC) formation in a TET-dependent manner [119,120]. In ESC, TET1 and TET2 were highly expressed, and high-capacity vitamin C increased the 5hmC level to four times the normal level after treatment, with 5fC and 5caC also being also increased by 10 to 20 times [119,121]. Severe deficiencies of TET2 and TET3 in HSPC lead to a loss of 5hmC of over 90%, leading to the spontaneous accumulation of DSB and further leading to the rapid progression of leukemia [122]. In addition, in human colon cancer cells, hematopoietic stem and progenitor cells (HSPCs), and leukemia cell lines, vitamin C treatment also leads to the induction of 5hmC levels and an increase in DNA hypomethylation throughout the entire genome [118,123,124,125,126,127,128]. Thus, these results demonstrate that vitamin C can modulate the epigenomic landscape via the direct modulation of TET enzyme function.

Furthermore, vitamin C deficiency cooperated with FLT3^ITD^ to accelerate leukemia progression in mice. When bone marrow cells from *Flt3^ITD^* mice were transferred into wild-type or ascorbate-depleted recipient mice due to the loss of gluconolactone oxidase (GULO)—the enzyme that produces vitamin C—the *Gulo*^−/−^ recipients developed a spectrum of disease from MPN to AML, whereas none of the control recipients exhibited any sign of malignancies [129]. Ascorbate depletion promoted 5hmC loss in TET2-deficient HSPCs, indicating that vitamin C depletion could further impair the activity of other TET proteins [125,129]. Restoring TET function by administering vitamin C promoted cell differentiation and cell death by attenuating DNA hypermethylation at tumor suppressor loci [125]. In addition, studies in several cancer cells have shown that vitamin C induced DNA hypomethylation by oxidizing 5mC in a TET2-dependent manner and increased endogenous retroviruses (ERVs) expression mimicking viral infection, thus inducing innate immune responses that enhanced the apoptosis of leukemia and solid tumor cell lines [124,130]. Given that most patients with hematologic neoplasms are *TET2* haplodeficient and that *TET1* and *TET3* mutations are rare, it is likely that activating the residual TET2 or the other TET family members TET1 and TET3 may be a promising treatment strategy for TET2-deficient malignancies [131,132]. Further studies are warranted to clearly define vitamin C’s clinical effect as well as its precise molecular basis in patients with TET-disrupted hematological malignancies.

### 4.3. Vitamin C in Cancer Therapy

Cancer patients were reported to have significantly reduced plasma vitamin C concentration compared with healthy people [109,124,133], and restoring vitamin C levels suppressed malignant cell growth (Table 2) [129]. In cancer cells, vitamin C promoted the reactivation of tumor suppressors, thereby reducing cell viability and altering sensitivity to treatments. For instance, the treatment of vitamin C in human skin and colon cancer cells promoted an increase in 5hmC and a decrease in 5mC in the CpG island on the promoter of p16INK4a and p21, which were often hypermethylated in cancer cells, resulting in the increased expression of those genes [134,135]. Vitamin C treatment in human leukemia cell lines and primary mice was also shown to induce 5hmC formation, DNA hypomethylation, and TET2/TET3-dependent gene expression involved in BER such as GADD45, PARP, and DNA glycosylase, consequently inhibiting abnormal self-renewal and the progression of cancer cells [125]. Vitamin C supplements have been considered supplements to existing cancer treatments. As plasma vitamin C levels decreased significantly in patients receiving chemotherapy, vitamin C treatment combined with standard chemotherapy could increase the effectiveness of chemotherapy [136]. The function of vitamin C in DNA methylation and tumor suppression has also been validated in subsequent studies using cancer cells in the kidneys, bladder, lungs, colon, and breast [127,128,137]. A reduction in 5hmC was associated with poor survival in renal cell carcinoma and vitamin C restored 5hmC levels via TET activation, significantly impeding tumor growth in vitro and in vivo [138,139]. Considering that TET enzymes play a primary role as tumor suppressors and many clinical effects of vitamin C seem to be mediated by TET proteins, incorporating vitamin C or other drugs specifically activating TET enzyme into the regimens for cancer treatments looks promising.

### 4.4. Combination Therapy

Several cell and animal models and recent clinical trials have demonstrated that high-dose vitamin C treatment in combination therapy reduces the viability of cancer cells and improves the therapeutic efficacy of existing chemotherapy. Ovarian cancer and glioblastoma patients receiving intravenous vitamin C, in combination with radiation therapy and temozolomide, displayed improved survival rates [150,151]. Moreover, vitamin C exhibited a synergistic effect in killing human AML cells when treated with PARP inhibitor olaparib [152].

In addition to its anti-cancer effects in immunocompromised preclinical models, vitamin C also exhibited clinical effects in immunocompetent animal models [153]. Recently, several mouse experiments confirmed that high-dose vitamin C enhanced the anti-cancer effect of immune checkpoint treatment [153]. In a mouse lymphoma model, vitamin C exhibited a significant synergistic effect with anti-PD1 therapy [154]. High-dose vitamin C increased the infiltration of immune cells, including CD4, CD8 T cells, and macrophages, into the tumor microenvironment and induced the production of granzyme B and IL-12 by CD8/natural killer cells and macrophages, respectively. Moreover, vitamin C enhanced the cytotoxic activity of adoptively transferred CD8 T cells, cooperated with anti-PD-1 and anti-CTLA4 treatments in mice bearing syngeneic tumors, and inhibited tumor growth in a T cell-dependent manner synergistically with anti-PD1 therapy [155,156]. TET2 augmented immunotherapy efficacy when targeted to INFγ target genes such as Th1-type chemokines and PD-L1 upon anti-PD-1 treatment and the loss of TET2 in tumors substantially reduced the effect of immunotherapy as well as its synergistic effects with vitamin C [153,155]. Although several studies have shown its dependence on TET proteins, it is not fully understood how vitamin C exhibits its anti-cancer effect. Furthermore, a requirement of high-dose administration in clinical trials and a lack of target specificity could restrict the application of vitamin C in the clinic. Thus, it is necessary to identify more specific TET agonists.

## 5. Metabolic Modulation of TET Function

### 5.1. IDH Inhibitors

The high frequency of *IDH1*/*IDH2* mutations and their potential roles in leukemogenesis accelerated the development of drugs specifically targeting mutant IDH1/IDH2. Ivosidenib (AG-120) and enasidenib (AG-221) are targeted inhibitors of mutant IDH1 and IDH2 enzymes, respectively (Figure 3 and Table 2) [140]. They were approved by the FDA for relapsed or refractory AML with mutant IDH based on phase 1/2 clinical trials and efficacy data, and their effects are still being assessed in other diseases including hematologic malignancies, glioma, cholangiocarcinoma, and chondrosarcoma. A precursor form of enasidenib was first identified from a high-throughput screening aimed at discovering selective allosteric inhibitors of the most prevalent form of mutant IDH2 in AML. It was shown to inhibit the conversion of α-KG to 2HG as expected by binding to the mutant IDH enzyme [142]. Enasidenib inhibited the growth of patient-derived blast cells and reversed the histone hypermethylation associated with the mutant IDH2. Ivosidenib, which competes with the enzyme’s cofactor magnesium ion and inhibits the formation of a catalytic site of the enzyme, is a reversible and allosteric inhibitor of mutant IDH1. Similar to enasidenib, the therapeutic effect of ivosidenib is attributable to 2HG suppression. Ivosidenib inhibited the oncogenic properties of cancer cells in AML myeloblasts and chondrosarcoma cells, which is also supported by phase I clinical trials for glioma patients [157,158]. It remains to be determined whether IDH inhibitors displayed clinical efficacy mainly via the activation of TET enzymes or other targets. Furthermore, there has been an assumption that minimal residual TET activity supports the survival and expansion of neoplastic HSPCs. Consistently, *IDH1/2* mutant-derived 2HG displayed striking synthetic lethality in TET-deficient cells. These results suggest that TET antagonists may also constitute a new class of therapeutic agents targeting *TET2* mutant neoplasms [149].

### 5.2. Succinate and Fumarate

Succinate and fumarate are the metabolic intermediates of the TCA cycle acting as the competitive inhibitors of many α-KG-dependent dioxygenases [143]. Mutations in *succinate dehydrogenase* (*SDH*) and *fumarate hydratase* (*FH*) were found in diverse cancers including paraganglioma, pheochromocytoma, and papillary renal carcinoma [144,159]. These mutations induced the accumulation of succinate and fumarate, respectively, up to millimolar levels and promoted cancer development possibly via two mechanisms: epigenetic modification and activation of hypoxic signals. By virtue of structural similarity relative to α-KG, both succinate and fumarate competitively inhibited α-KG-dependent dioxygenases that were involved in epigenetic regulation, including TET proteins (Figure 3 and Table 2). They decreased the global 5hmC levels in neuroblastoma cells and the expression of HIF target genes via TET inhibition [160].

### 5.3. Itaconate (ITA)

*Immune responsive gene 1* (*IRG1*), also known *cis-aconitic acid decarboxylase* (*CAD*), encodes a mitochondrial metabolic enzyme known as aconitate decarboxylase 1 (ACOD1) that catalyzes the decarboxylation of cis-aconitate to produce anti-inflammatory metabolite itaconic acid [161]. A recent study has shown that ITA exhibited its anti-inflammatory property by acting as a potent inhibitor of the TET enzyme. ITA directly bound to TET2 and potently inhibited TET activity (Figure 3 and Table 2) [145]. Lipopolysaccharide (LPS) treatment induced IRG1/ACOD1 expression and ITA accumulation in macrophages, concomitantly inhibiting TET activity. Transcriptome analyses have shown that TET2 was a major target of ITA in inhibiting LPS-induced genes, including those regulated by the NF-κB and STAT signaling pathways. ITA also decreased the levels of 5hmC in vivo depending on the catalytic activity of TET2, reduced acute pulmonary edema caused by LPS and tissue (i.e., lung and liver) injury, and protected mice from fetal endotoxemia. Collectively, these results suggest that the IRG1–ITA–TET2 axis may play a key role in broad physiological and pathological processes. It will be important to analyze the physiological function of IRG1 as a TET inhibitor in cancer therapy in future studies.

### 5.4. BCAA Transaminase 1 (BCAT1)

The branched-chain amino acid (BCAA) pathway has recently been associated with aggressiveness in several cancers [162,163]. A recent study has shown that BCAT1, which transfers α-amino groups from branched-chain amino acids (BCAA) to α-KG, causes the DNA hypermethylation phenotype in AML stem cells by reducing α-KG levels. By employing proteomic analysis, it was uncovered that BCAT1 is overexpressed, and the BCAA pathway is enriched in leukemia stem cells [164]. Depleting BCAT1 impaired the growth and leukemia-initiating potential of AML cells. HIF1α overexpression restores cell growth and survival after BCAT1 knockdown. Mechanistically, BCAT1 overexpression mimicked the effect of IDH mutations by reducing intracellular α-KG concentration. Consistent with the fact that α-KG is a key cofactor for EGL-9 family hypoxia-inducible factor 1 (EGLN1) and TET enzymes, BCAT1 depletion increased levels of α-KG, leading to EGLN1-mediated HIF1α degradation, while its overexpression diminished α-KG levels and thereby induced DNA hypermethylation by inhibiting TET2 (Figure 3 and Table 2). Moreover, high BCAT1 expression was associated with the enrichment of leukemia stem-cell signature genes, and BCAT1 expression increased upon AML recurrence. Thus, these results suggest that the BCAA-BCAT1-α-KG axis modulating α-KG-dependent enzymes including TETs is a new therapeutic target for AML patients [164].

## 6. Other TET Modulators

### 6.1. AMP-Activated Kinase (AMPK)

AMPK is a heterotrimeric protein complex that senses the concentration of ATP, ADP, and AMP, and it regulates whole-body energy balance [165]. TET2 is phosphorylated at Ser99 by AMPK, stabilizing TET2 proteins and levels of 5hmC (Figure 3 and Table 2) [141]. AMPK activator metformin activated AMPK and thus elevated the total TET2 levels in hyperglycemic mice, significantly reducing tumor growth in vitro and in vivo. These data indicated that AMPK activators can be anti-cancer drugs influencing the AMPK-TET2-5hmC axis by stabilizing TET2 proteins. However, it remains to be determined whether these drugs would exhibit efficacy in patients.

### 6.2. Silence Information Regulator 1 (SIRT1)

TET2 was shown to be acetylated at K110 by histone acetyltransferase p300, which resulted in enhanced enzymatic activity and stability while HDAC1/2 reversed this process [166]. However, it remains to be determined whether the pharmacological activation of p300 may elicit clinical benefits in cancer treatments. SIRT1 is a nicotinamide adenine dinucleotide (NAD^+^)-dependent histone deacetylase that has pleiotropic effects on various cellular processes, including cell proliferation, differentiation, metabolism, inflammatory immune response, oxidative stress, apoptosis, and other processes [167,168]. Recently, a study using HSPCs in MDS patients has shown that SIRT1 is a critical regulator of TET2 protein levels (Figure 3 and Table 2). In these cells, SIRT1 was downregulated via mIR-9 and miR-34a. SIRT1deficiency promoted the growth of HSPCs and their self-renewal [56]. Interestingly, SIRT1 was bound to a conserved lysine residue in the TET2 catalytic domain and deacetylates TET2, thus enhancing its enzymatic activity. Consistently, in the human MDS xenograft model, SIRT1 agonists inhibited HSPC self-renewal and engraftment capacity via TET activation. Thus, this work has suggested the therapeutic potential of SIRT1 agonists for MDS treatment via the restoration of TET2 function. However, in a Parkinson’s disease model, SIRT1 was shown to promote deacetylation and the proteasomal degradation of TET2. Thus, more studies are necessary to define the precise impact of SIRT1 modulation on TET2 protein [169].

### 6.3. Other Potential TET Antagonists (Figure 3 and Table 2)

Dimethyloxalylglycine (DMOG), an ester of N-oxalyglycine, is a small molecule that has a similar structure to α-KG and potentially functions as an inhibitor of TET proteins [146]. During embryo development, DMOG was shown to inhibit TET3, leading to elevated methylation and the suppressed expression of pluripotency factors such as NANOG and OCT4 [170]. Furthermore, in an assay for identifying cytosine-based TET antagonists, Bobcat339 was identified as the most potent TET inhibitor, displaying effects against TET1 and TET2, but not DNMT3a, and reducing 5hmC levels in the DNA of cultured neuron cells [171]. However, the inhibitory effect of Bobcat339 is controversial in that a recent study has shown that the original inhibitory effect is a result of contaminating copper (II), and it has no effect on human TET1/TET2 [147].

Additionally, the virtual ligand screening of natural products further identified compound 35 (termed C35), a catechol-containing small molecule, as a novel TET inhibitor. C35 targeted the catalytic core of TET enzymes without affecting α-KG binding or its activity [148]. C35 treatments reduced the levels of TET-mediated 5hmC in vitro and increased the efficiency of somatic cell programming. Moreover, via in silico docking using the cocrystal structure of the TET2 catalytic domain conjugated with N-oxalylglycine (NOG), a non-specific inhibitor of α-KG-dependent enzymes, TETi76 was identified as an α-KG-competitive and highly selective TET1/2/3 inhibitor [149]. TETi76 mimicked the loss of TET activity and downregulated cellular 5hmC levels, potently antagonizing the clonal expansion of TET2-deficient cells. Similarly to 2HG, TETi76 also induced synthetic lethality in TET-deficient human leukemia cells in vitro and in vivo [149].

## 7. Conclusions and Future Perspectives

Over the past few decades, DNA (de)methylation has been highlighted as a fundamental epigenetic mechanism impacting both normal cellular physiology and neoplastic transformation. DNA methylation and demethylation dynamics are tightly regulated by the intricate interplay between DNMT and TET enzymes to maintain normal cellular differentiation and functions. It is also critical for securing the barrier between normal and cancerous states. A number of previous studies using various cell lines, animal models, and clinical samples point to the promising potential of targeting the epigenetic regulatory function of DNMT and TET enzymes for cancer treatment. Several small molecules, including some DNMT inhibitors or TET activators/inhibitors, are being used or tested for their efficacy as novel anticancer drugs. Nonetheless, there are challenges that need to be clearly resolved in future research, including the lack of target specificity, molecular mechanisms underlying the therapeutic efficacy of each drug, the molecular basis of drug resistance, possible side effects, potential synergism with conventional therapies, etc. Thus, further studies are required to develop more effective and specific epigenetic therapies that apply to a broad range of cancers.

## Figures and Tables

**Table 1:** DNMT inhibitors in the clinical trial.

Group	Mechanisms of Action	Drug	Target(s)	Disease(s) and Phase Studies	References
Nucleoside analogs	Incorporate into DNA instead of cytidine; associate with DNMT1; induce DNMT degradation and DNA demethylation.	5-azacitidine (5-AZA)	DNMT1	MDS and AML (FDA-approved); glioma, prostate cancer, pancreatic cancer, ovarian cancer, metastatic melanoma	[38,39,40,41]
		5-aza-2′-deoxycytidine (decitabine)	DNMT1	MDS and AML (FDA-approved); CML, prostate cancer, thyroid cancer	[39,42,43]
		Zebularine	DNMT1	MDS, solid tumors	[44]
		Guadecitabine (SGI-110)	DNMT1	AML, MDS, and HCC (phase II); CMML, ovarian cancer, urothelial carcinoma, colorectal cancer, peritoneal cancer	[45]
		4′-thio-2′-deoxycytidine (TdCyd)	DNMT1	Refractory solid tumors	[46]
		5-fluoro-2′-deoxycytidine (FdCyd)	DNMT1	AML, MDS, head and neck tumors, lung tumors, urinary bladder tumors, breast tumors	[47]
Non-nucleoside analogs	Block the catalytic site of DNMTs; induce DNA demethylation.	Procainamide	DNMT1	Prostate cancer, breast cancer, colon cancer, and non-small-cell lung cancer	[48]
		Procaine	DNMT1, DNMT3A	Breast cancer, gastric cancer, hepatocellular carcinoma, and non-small-cell lung cancer cells	[49]
		Nanaomycin A	DNMT3B	Colon cancer, lung cancer, and bone marrow cells	[50]
		Hydralazine	DNMT1	Ovarian cancer, cervical cancer, refractory solid tumors, breast cancer (phase II).	[51]
		MG98	DNMT1	Solid tumors (phase I)	[52,53]
		N-phthaloyl-L-tryptophan (RG108)	DNMT1	Solid tumors	[54]
		Disulfiram	DNMT1	Refractory multiple myeloma, prostate cancer (phase II)	[55]
		SGI-1027	DNMT1, DNMT3A/B	Hematological cancer, solid tumors	[56]
		GSK3685032	DNMT1	AML	[57]

**Table 2:** List of known TET activators and inhibitors.

Group	Drug	Target(s)	Effect(s)	Disease(s)	References
TET activators	Vitamin C	TET1/2/3	Increases 5hmC levels via TET activation; induces DNA hypomethylation	MDS, AML	[125,129]
	AG-120 (ivosidenib), AG-221 (enasidenib)	mIDH	Reduce the accumulation of 2HG; recover TET-dependent DNA demethylation	AML, hematologic malignancies, glioma, cholangiocarcinoma, chondrosarcoma	[140]
	SRT1720 (SIRT1 agonist)	TET2	SIRT1 agonist; deacetylates TET2 at conserved lysine in its catalytic domain; enhances TET2 activity	MDS	[56]
	AMPK activators (AICAR, Metformin, A769662)	TET2	Phosphorylates TET2; maintains stability of TET2; induces levels of 5hmC	Hyperglycemia-related tumor	[141]
TET inhibitors	2-Hydroxyglutarate (2HG)	TET2	Induces DNA hypermethylation, gene silencing, and tumor progression	Hematological malignancies, AML, MDS	[142]
	Fumarate	TET1/2/3	Downregulates 5hmC levels; induces DNA hypermethylation	Hematological malignancies, AML, MDS	[143]
	Succinate	TET1/2/3	Downregulates 5hmC levels; induces DNA hypermethylation	Hematological malignancies, AML, MDS	[144]
	Itaconate	TET2	Competes with α-KG to inhibit TET2; dampens LPS-induced inflammatory responses	Hematological malignancies	[145]
	Dimethyloxallyl glycine (DMOG)	TET3	Increases 5mC levels; downregulates pluripotency genes	Solid tumors	[146]
	Bobcat339	TET1/2	Inhibits TET activity; reduces 5hmC levels	In vitro	[147]
	C35	TET1/2/3	Inhibits TET activity and somatic cell reprogramming	In vitro	[148]
	TETi76	TET1/2/3	Reduces 5hmC levels; growth inhibition of TET-deficient leukemic cells	Hematological malignancies, MDS, AML	[149]

## Data Availability

Not applicable. No new data were created or analyzed in this work.
